# Learning and Memory Processes Following Cochlear Implantation: The Missing Piece of the Puzzle

**DOI:** 10.3389/fpsyg.2016.00493

**Published:** 2016-04-08

**Authors:** David B. Pisoni, William G. Kronenberger, Suyog H. Chandramouli, Christopher M. Conway

**Affiliations:** ^1^Speech Research Laboratory, Department of Psychological and Brain Sciences, Indiana UniversityBloomington, IN, USA; ^2^Riley Child and Adolescent Psychiatry Clinic, Department of Psychiatry, Indiana University School of MedicineIndianapolis, IN, USA; ^3^NeuroLearn Lab, Department of Psychology, Georgia State UniversityAtlanta, GA, USA

**Keywords:** learning, memory, repetition, free-recall, semantic clustering

## Abstract

At the present time, there is no question that cochlear implants (CIs) work and often work very well in quiet listening conditions for many profoundly deaf children and adults. The speech and language outcomes data published over the last two decades document quite extensively the clinically significant benefits of CIs. Although there now is a large body of evidence supporting the “efficacy” of CIs as a medical intervention for profound hearing loss in both children and adults, there still remain a number of challenging unresolved clinical and theoretical issues that deal with the “effectiveness” of CIs in individual patients that have not yet been successfully resolved. In this paper, we review recent findings on learning and memory, two central topics in the field of cognition that have been seriously neglected in research on CIs. Our research findings on sequence learning, memory and organization processes, and retrieval strategies used in verbal learning and memory of categorized word lists suggests that basic domain-general learning abilities may be the missing piece of the puzzle in terms of understanding the cognitive factors that underlie the enormous individual differences and variability routinely observed in speech and language outcomes following cochlear implantation.

## Introduction

For a number of years, my colleagues and I have been on a mission to understand and explain the reasons for the enormous individual differences and variability in speech and language outcomes following cochlear implantation in adults and children. In numerous papers, we have argued that the individual differences routinely observed at all implant centers around the world are not mysterious, anomalous or idiopathic in nature but instead reflect differences and natural sources of variability in more basic elementary building blocks of cognition (Pisoni et al., 2008). These cognitive factors include the early registration, sensory encoding, storage, rehearsal, retrieval, and processing of phonological and lexical representations of spoken words in speech perception and spoken language processing tasks. In our search for underlying process-based explanations of individual differences, we have focused our research efforts on issues related to learning and memory, two central topics in cognition that have been neglected in the field of cochlear implantation.

This paper is organized into six main sections. In the first section (The Puzzle about Outcomes following Cochlear Implantation), we introduce and discuss the longstanding problem of variability in speech and language outcomes following cochlear implantation and suggest that learning, memory, and related cognitive processes may represent the missing piece of the puzzle to understanding such variability. In the next two sections (“Explicit Sequence Memory Spans” and “Explicit Sequence Learning Spans”), we present a summary of some of our earlier research findings showing atypical explicit memory and learning of auditory and visual serial patterns in deaf children with cochlear implants (CIs; Pisoni and Cleary, 2004). We next review an area of research investigating the “Hebb repetition effect” that provides additional evidence for understanding deficits in serial memory and learning and language outcomes (The “Hebb Effect” and Sequence Repetition Learning). In the Section “Implicit Learning of Sequential Patterns,” we review research demonstrating that deaf children with CIs show atypical implicit learning of sequential patterns, and this disturbance may be part of the reason for the observed language delays (Conway et al., 2011b). Finally, in the Section “Verbal Learning and Memory Processes,” we describe some recent findings on verbal learning and memory in prelingually deaf long-term CI users obtained with the California Verbal Learning Test (CVLT-II; Delis et al., 2000), a well-known and widely used neuropsychological assessment instrument that provides information about the control processes and organizational strategies that individuals use in free recall of categorized word lists (Chandramouli et al., Manuscript in preparation). We discuss the theoretical and clinical implications of all of these findings in the “Theoretical and Clinical Implications” Section of this paper. Overall, we suggest that the broad domain of learning and memory may turn out to be a very important aspect of cognition that provides a principled explanation for the enormous individual differences routinely observed in outcomes following implantation.

## The Puzzle About Outcomes Following Cochlear Implantation

The variability and individual differences observed following cochlear implantation in profoundly deaf adults and children is enormous and represents a significant clinical problem in the field of otology and audiology. At the present time, there is no question that CIs work and often work very well in quiet listening conditions for many profoundly deaf children and adults. The speech and language outcomes data published in the clinical and basic science journals over the last two decades document quite extensively the clinically significant benefits of CIs using numerous behaviorally based outcome measures of speech recognition, speech intelligibility, and language processing in both children and adults who have received these sensory aids as a medical treatment for their profound deafness. Without a CI, a prelingually deaf infant or a young child with a profound bilateral hearing loss would not be able to acquire receptive and expressive spoken language skills and would display significant global developmental and intellectual delays that would remain over his/her entire lifetime.

Recognizing these successes, Wilson et al. (2011, p. 117) stated recently that CIs represent “one of the great success stories of modern medicine” and that “the CI is the most successful neural prosthesis developed to date” and “exceeds by orders of magnitude the number for all other types of neural prostheses.” Despite these recent broad sweeping statements about the “efficacy” of CIs as a medical intervention for profound hearing loss in both children and adults, there still remain a number of challenging unresolved clinical and theoretical issues that deal with the “effectiveness” of CIs in individual patients that have not been successfully resolved yet despite many years of basic and clinical research (Pisoni et al., 2008).

After receiving a CI, all patients require a relatively long period of sensory acclimatization and perceptual adaptation to learn how to process underspecified acoustic-phonetic information encoded in the degraded signal. This sensory and perceptual adaptation must occur before these patients are able to derive any functional benefits from their implant and display solid evidence of perceiving speech, understanding spoken language, and reliably recognizing natural environmental sounds. Despite the importance of learning and adaptation following implantation, this particular domain of cognition has received little attention compared to the voluminous literature on conventional speech perception and language outcomes.

The revolution in the field of experimental psychology in the 1960s that gave birth to the new field of cognitive psychology has had a profound and long-lasting influence on our thinking about how humans perceive, encode, store, and process information (Neisser, 1967; Haber, 1969). Armed with new experimental methods and a richer and more powerful theoretical conceptualization of how these complex cognitive processes might be carried out by humans, the field of cognition has flourished over the last 50 years and has made important contributions to many related fields including neuroscience, developmental and clinical science, and social psychology. Until recently, the cognitive approach has been very slow to have a significant impact in the field of clinical audiology and, in particular, research on hearing loss and CIs which has been heavily dominated by the medical community of otologists, audiologists, and speech-language pathologists (Arlinger et al., 2009; Jerger, J. cited in Fabry, 2011, p. 20).

While there have been several small steps made applying some of the methods and theory of information processing psychology to problems in CIs (Pisoni, 2000), one of the core foundational areas of cognition that has been neglected in almost all of the clinical research on CIs in both adults and children is learning and memory processes and the organizational strategies that CI users employ in conventional laboratory-based episodic memory and learning tasks such as free-recall, recognition, and repetition-based learning. The precise reasons for the lack of research on these central topics are unclear at this time but the current situation may simply reflect the long-standing historical biases toward the study of peripheral sensory processes in the field of hearing research and clinical audiology and the strong reluctance over the years to fully acknowledge that hearing loss in the pediatric population is primarily a “brain” issue and not an “ear” issue (Luria, 1973; Flexer, 2011). The absence of a large body of basic research on learning and memory processes in deaf children and adults with CIs represents a very significant gap in our basic knowledge and understanding of cognition, neural plasticity, and experience- and activity-dependent learning, core foundational processes which underlie all adaptive behaviors in both humans and animals.

Although our focus in this paper is on the role of cognition, specifically, learning and memory processes, we do not want to minimize in any way the important contributions of demographics and other contributing factors to speech and language outcomes following implantation. The evidence collected over the years linking outcomes to variables like age of implantation, communication mode, family and device factors as well as a host of audiological and hearing-related variables is very strong and reliable. However, these factors taken in isolation fail to account for a significant part of the variance observed in the conventional speech and language outcome measures routinely obtained from CI users at centers around the world. Additional sources of variance, we argue, come from cognitive factors such as learning, memory, attention, inhibitory control, working memory, executive function, and cognitive control processes. Our point in this paper is that demographics do not fully account for the whole story and in many cases may obscure more basic underlying elementary processes. Moreover, the focus on demographics and conventional endpoint measures of outcome and benefit often prevents researchers from moving beyond descriptive accounts to explanatory causal explanations framed within a broader theoretical context that emphasizes the role of basic elementary information processing operations, such as learning and memory processes.

In the remaining sections of this paper, we summarize some of the major findings from our ongoing program of research on learning and memory processes following cochlear implantation and discuss the broader clinical and theoretical implications of these findings for understanding the factors underlying individual differences and variability in speech and language outcomes.

## Explicit Sequence Memory Spans

**FIGURE 1:**
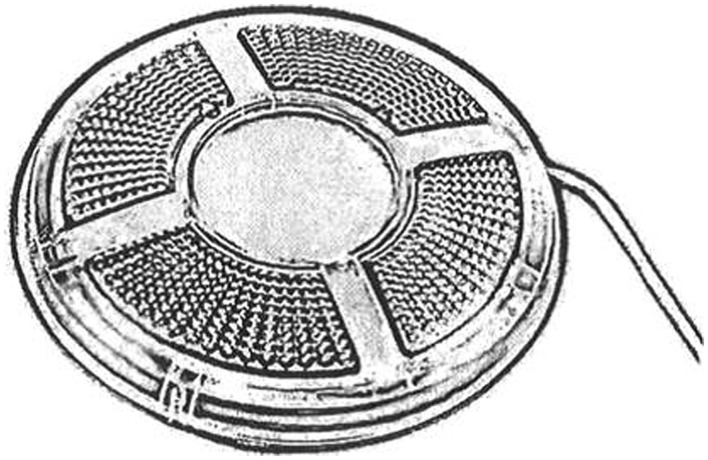
**Sequence memory response box based on Milton–Bradley’s original Simon game.** [Adapted from earlier studies carried out by Cleary et al. (2001) and Pisoni and Cleary (2004)].

The traditional methods for measuring immediate memory capacity using digit spans require a subject to encode both item and order information and then verbally repeat back and reproduce the sequence of test items using an overt articulatory motor response (Dempster, 1981). Because most deaf children with CIs also have other comorbid delays in speech development and often display “atypical” articulation and speech motor control because of their early hearing loss, it is possible that any differences observed in auditory short-term memory or working memory tasks using conventional digit span tests could be due to the nature of the response organization requirements during retrieval and response output processes in addition to any possible differences in early sensory registration, encoding, storage, or retrieval processes (AuBuchon et al., 2015). To eliminate the use of an overt articulatory-verbal motor response, we developed a new experimental methodology to measure sequence memory based on Milton–Bradley’s Simon ©, a well-known memory game that uses a simple reproduction task. **Figure 1** shows a display of the apparatus we used in our early studies (Cleary et al., 2001; Pisoni and Cleary, 2004). We took an off-the-shelf Simon memory game box and modified it in our shop by building a custom interface to a PC so we could directly control the stimulus presentation, record the subject’s responses, and provide feedback when needed.

In our version of the Simon sequence memory task, a child hears or sees a sequence of color names or color lights presented by the computer and then simply “reproduces” the stimulus pattern by depressing a sequence of colored response panels on the four-alternative Simon response box using a manual response. Because the Simon memory game was controlled by a computer, we were able to manipulate the stimulus presentation conditions in several different ways while also holding the response format constant. In addition to measuring sequence memory spans, the Simon memory game apparatus and methodology also provided us with an opportunity to study basic learning processes, specifically, serial learning and the relations between sequence memory and serial learning using the same experimental procedures and same response demands (Cleary et al., 2001; Karpicke and Pisoni, 2004; Pisoni and Cleary, 2004).

The lights on the Simon apparatus were illuminated in temporal patterns from a vocabulary ensemble of four colors. Before the memory game began, we asked each child to identify recorded audio tokens of the four color-names by pointing to one of the four large colored buttons on the response box to make sure they could hear and recognize the four color names without any errors. Three types of sequential patterns were presented in separate blocks: auditory-only (A-only), lights-only (L-only), and auditory+lights (A+L). All of the sequences used for the memory game task were generated pseudo-randomly by a computer program from the four alternative colors, with the stipulation that no color name or color light would be repeated consecutively in any given list. Each subject started with a list length of one item. If two sequences in a row at a given list length were correctly reproduced, the next sequence that was presented was increased in length by one item that was chosen randomly from the four colors. If the list was incorrectly reproduced on any trial, the next trial presented a new list that was one item shorter in length. This up-down adaptive tracking procedure is similar to methods typically used in psychophysical testing (Levitt, 1970). Importantly, novel sequences were generated randomly on each trial in order to prevent any learning from occurring other than routine practice effects that would typically be observed in learning how to carry out a new task in an unfamiliar laboratory setting.

**FIGURE 2:**
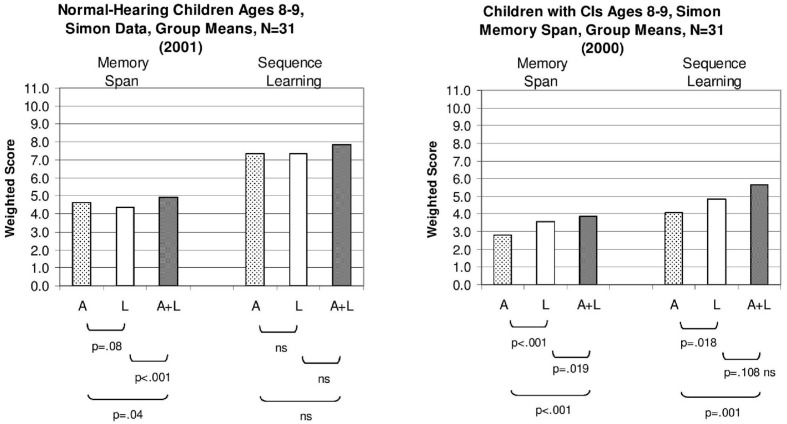
**Mean sequence memory span scores and sequence learning span scores for two groups of children tested using the “Simon” memory game.** Scores for the group of normal-hearing 8- and 9-year-old control children are shown on the **(Left)**; scores for the group of 8- and 9-year old deaf children with cochlear implants (CIs) are shown on the **(Right)**. Speckled bars represent mean weighted span scores in the auditory-only condition (A-only), open bars represent span scores in the lights-only condition (L-only), and shaded bars indicate scores in the auditory-plus-lights condition (A+L). For each task, *p*-values for paired *t*-tests between the conditions are provided. [Adapted from data reported by Pisoni and Cleary (2004)].

We computed a “weighted” sequence span score for each child which was calculated by finding the proportion of lists correctly reproduced at each list length and summing these proportions across all list lengths (Pisoni and Cleary, 2004). A summary of the results from the Simon sequence reproduction memory span task for two groups of 8–9 year-old-children is shown in **Figure 2**. The weighted-span scores for the normal-hearing aged-matched children (*N* = 31) are shown in the left panel while the scores for the deaf children with CIs (*N* = 31) are shown in the right panel. Within each panel, the scores for A-only presentation condition are shown on the left, scores for L-only presentation are shown in the middle and scores for the combined A+L condition are shown on the right.

Examination of the sequence memory span scores revealed several important differences between the two groups. Not surprisingly, the sequence memory spans for the A-only and A+L presentation conditions were consistently lower overall for the children with CIs than the normal-hearing children. However, the deaf children with CIs displayed shorter sequence memory spans in the L-only condition than the normal-hearing children. This was an unexpected and surprising finding that provides additional converging support for the hypothesis that rapid phonological recoding and efficient verbal rehearsal processes in short-term working memory play an important inseparable role in perception, learning, and memory in these children (Pisoni and Geers, 2000; Pisoni and Cleary, 2004). Capacity limitations of verbal short-term memory are closely tied to speed of processing information even for visual sequential patterns that can be rapidly recoded and rehearsed in verbal short-term memory using a phonological or articulatory code in sequential processing tasks (Conrad, 1960). Verbal coding strategies may be mandatory or at least commonly used by humans who are engaged in memory tasks that require immediate serial recall (ISR) of patterns that preserve item and order information (Gupta and MacWhinney, 1997). Although the visual patterns were presented using only sequences of colored lights, many of the participants, particularly the normal-hearing children, likely recoded the serial patterns using well-learned automatized verbal labels and coding strategies in order to create stable representations of the stimulus patterns in working memory for maintenance and rehearsal prior to response organization and motor output.

When compared to the group of normal-hearing controls, the deaf children with CIs may have used a different encoding strategy and less efficient verbal rehearsal processes for maintaining temporal sequences of the color name codes in working memory. Early auditory deprivation and the absence of sound stimulation following a period of prelingual profound hearing loss during the initial stages of language development may not only affect early sensory processing and perception but may also influence subsequent encoding and rehearsal processes in verbal working memory (Conrad, 1979). The deaf children with CIs in this study showed a reduced capacity to maintain serial order information in short-term memory even when the information was presented through the visual sensory modality (see Myklebust and Bruton, 1953). These findings on immediate sequence memory spans for auditory and visual patterns obtained with the Simon memory game which did not require any overt verbal articulatory-motor responses replicate the earlier memory span results we obtained using the WISC digit spans which showed large and consistent differences in memory span between deaf children with CIs and age-matched normal-hearing children (Pisoni and Geers, 2000; Pisoni and Cleary, 2004). To our knowledge, these were the first memory span data collected from prelingually deaf children with CIs demonstrating differences in immediate memory capacity and rehearsal processes without relying on any articulatory-based verbal response for output.

## Explicit Sequence Learning Spans

The initial version of our Simon memory game used novel sequences of color names or colored lights on each trial to measure immediate memory spans. As previously mentioned, all of the test sequences were generated randomly in order to prevent any learning from occurring other than routine practice effects. The primary goal of the first phase of this project was to obtain estimates of immediate memory capacity for serial patterns that were not influenced by repetition effects or idiosyncratic verbal coding strategies that might increase memory capacity from trial to trial (Cleary et al., 2001, 2002). There was no basis for any new learning to take place and the measures of Simon sequence memory span could be used to estimate the capacity of immediate memory for serial patterns of familiar color names or color lights.

In the second phase of this study, we used the same basic Simon memory game apparatus and procedure to study learning and to investigate the effects of sequence repetition on coding and rehearsal strategies in immediate memory. To accomplish this goal and to directly compare the gains in repetition learning and the increases in working memory capacity to our earlier sequence memory span measures, we examined the effects of repetition on immediate memory span. To measure learning, during a block of trials, the same visual or auditory pattern was repeated over again after each correct trial. Each new test sequence then increased in length by one item until the child was no longer able to reproduce the repeated pattern correctly anymore. This small procedural change in generating the test sequences provided an opportunity to measure sequence learning following repetition and to explore how sequence repetition affects the capacity of immediate memory (see Hebb, 1961). Everything else remained exactly the same as in the original sequence memory conditions except that the same serial pattern was repeated after each correct reproduction.

The right-hand bars in **Figure 2** display a summary of the results obtained in the Simon learning conditions that investigated the effects of stimulus repetition on sequence learning spans. The same three presentation formats used in the earlier sequence memory conditions were used again, that is, A-only, L-only, and A+L. The weighted memory span scores for the sequence memory conditions obtained earlier under random presentation in the first phase are shown by the left-hand bars of each panel in **Figure 2**; the corresponding set of sequence learning span scores obtained following repetition for the same three presentation conditions are reproduced on the right-hand side of each panel. The data for the 8- and 9-year-old normal-hearing children are shown in the left panel; the data for the 8- and 9-year-old deaf children with CIs are shown in the right panel.

Examination of the two sets of sequence span scores shown within each panel reveals several consistent findings. First, just repeating the same stimulus sequence again after a correct reproduction produced robust repetition learning effects for both groups of children. This sequence repetition effect can be seen clearly by comparing the three scores on the left-hand side of each panel to the three scores on the right-hand side. In every case, the sequence learning span scores on the right are higher than the sequence memory span scores on the left. Repetition of a serial pattern increased immediate memory span capacity although the magnitude of the improvement differed across the two groups of subjects. Although a sequence repetition effect was also obtained with the deaf children who use CIs, the size of their improvement was about half the size of the repetition effect found for the normal-hearing children shown in the left-hand panel. Second, the rank ordering of the three presentation formats in the sequence learning conditions was similar to the rank ordering observed in the sequence memory span conditions for both groups of children. The repetition effect was always largest for the A+L conditions for both groups due to redundancy gains when both modalities are combined together.

**FIGURE 3:**
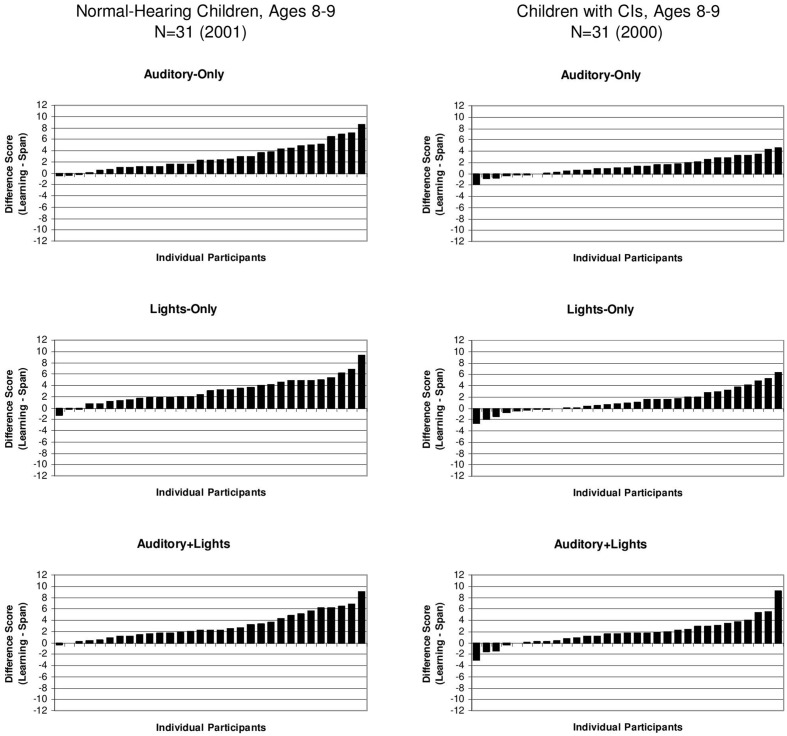
**Difference scores for individual subjects showing a child’s sequence learning span score minus his/her sequence memory span score.** Scores for the A-only are shown on the **(Top)** of each panel, scores for the L-only condition are shown in the **(Middle)** panel and scores for the A+L condition are shown in the **(Bottom)** panel. Scores for the normal-hearing 8- and 9-year-old children are shown on the left and scores for the 8- and 9-year-old deaf children with CIs are shown on the right. [Adapted from data reported by Pisoni and Cleary (2004)].

To assess the magnitude of the sequence repetition learning effects for the individual children in both groups, we computed difference scores between the learning and memory conditions by subtracting the memory span scores from the learning span scores for each subject. The difference scores for all of the individual subjects in both groups for the three presentation formats are displayed in ascending order in **Figure 3**. Scores above zero in the **Figure 3** indicate the presence of a repetition benefit; scores below zero indicate no repetition learning. Inspection of these distributions reveals a wide range of individual performance for both groups of subjects. Some subjects showed relatively large gains in learning while others showed only very small gains. Although most of the subjects in both groups displayed some evidence of repetition-based sequence learning in terms of showing a positive repetition effect, there were a few subjects at the end of the distribution who either failed to show any sequence repetition learning effect at all or showed a small reversal of the predicted repetition effect.

While the number of these subjects was quite small in the group of normal-hearing control children, we found that about one-third of the deaf children (*N* = 11) showed no evidence of a sequence repetition learning effect at all and obtained no benefit from having the same stimulus sequence repeated over again on each trial. The failure of a large subset of deaf children with CIs to display any evidence of simple repetition-based sequence learning following presentation of a visual pattern in the reproduction memory task suggests the presence of a significant impairment in serial learning for both auditory and visual patterns.

As explained previously, given the nature of the stimuli used in the Simon task, it is likely that the normal hearing children were using a verbal rehearsal strategy to label and help remember each color sequence as it occurred (e.g., “RED–BLUE–GREEN–BLUE” etc.). It is possible that the reduced sequence memory and learning spans in the deaf children with CIs is due to atypical verbal rehearsal or even a non-verbal coding strategy. In order to tease apart the extent that the sequence memory and learning impairments were due to atypical verbal rehearsal strategies, we recently designed a new version of the Simon sequence learning and memory task using a touch-screen monitor that incorporated four different conditions to assess both verbal and non-verbal visual sequencing (Gremp et al., Manuscript in preparation). Two of the conditions used black and white visual stimuli instead of colored squares in order to make verbal rehearsal less likely. In addition, half of the tasks used sequences that were randomly generated on each trial, as was the case in the first set of studies described above to assess sequence memory, while the other half used repeating sequences on each trial to measure sequence repetition learning effects. Thus, this design provided a direct way to assess the effect of verbal coding on sequence memory and sequence learning. A group of deaf children with CIs and an age-matched group of normal-hearing children participated in the study. The findings revealed that while the deaf children with CIs showed lower performance for the verbal versions of sequence memory and sequence learning, their performance was lower overall on all versions of the task, regardless of whether verbal rehearsal was likely to have occurred. These recent findings suggest that the impairment on visual sequence memory and learning is not solely due to difficulties with verbal coding and verbal rehearsal but may reflect a more global domain-general disturbance in the learning and memory of sequential patterns.

Repetition-based learning of serial patterns like the learning and memory of highly familiar color sequences and visual-spatial patterns observed in this study is one of the earliest and most primitive forms of learning and adaptive functioning that the brain and nervous system carry out in acquiring knowledge and recording experiences about regularities in the surrounding environment. These are theoretically important findings in this clinical population because they link the present set of serial learning results to an extensive and rapidly growing literature on ISR, the Hebb repetition learning (HRL) effect and the learning of phonological word-forms and lexical development discussed in the next section.

## The “Hebb Effect” and Sequence Repetition Learning

The findings reported in the previous section on repetition effects in sequence memory and learning of serial patterns in deaf children with CIs using the Simon memory game methodology are closely related to a large body of research carried out on processing of serial order information and sequence learning and memory using an experimental methodology first developed by Donald Hebb more than 50 years ago (Hebb, 1961). In his seminal study on the effects of sequence repetition on serial learning and memory, Hebb gave 40 college subjects 24 randomized lists each containing nine randomized digits for ISR. An example of one of the lists he used is 591437826. The experimenter read the sequence of nine digits aloud to each subject at a rate of about one digit per second. Interspersed within these random lists of digits was one list of digits that was repeated over again after every third trial. Subjects were told to listen carefully to each list and simply repeat back the list of digits after presentation in the same order they were presented. The subjects were told that the purpose of the study was to see if memory span for sequences of random digits would improve with practice.

Hebb found that although his subjects showed no evidence of any improvement in reproduction of the randomized digit lists over the course of the experiment, they did show a very consistent pattern of learning and improvement for the repeated digit lists. After the experiment was over, Hebb asked his subjects if any of them noticed the repeating pattern. About half of the subjects stated that they were aware that some of the patterns repeated. Fifteen of the subjects stated that they had not observed any repetitions at all.

The improvement in performance following presentation of a repeating sequence of stimuli is known as the “Hebb Effect” in the human learning and memory literature, and this form of repetition-based sequence learning has recently taken on a special status in a series of novel studies on serial learning and phonological word-form learning (Page and Norris, 2009a). The HRL effect is a very robust finding that has been replicated and extended by Melton (1963), McKelvie (1987), and Stadler (1993) among others over the years. Hebb originally suggested that this “rather simple-minded experiment” provided strong evidence for the conclusion that a single repetition of a sequence of random digits could produce a permanent structural change in long-term memory without feedback and that this structural memory trace had fundamentally different properties from the sensory-based activity-traces that support short-term memory span (Hebb, 1961).

Although the HRL effect has received some modest amount of attention over the years since it was first reported, current interest in the HRL effect has accelerated quite rapidly over the last 10 years as several researchers lead primarily by Page and Norris (2009a,b) have recognized the potential usefulness of the experimental methodology and findings in the study of serial learning and sequence memory effects. In a series of recent papers, Page and Norris (2009a,b) have extended and elaborated Hebb’s original findings on repetition-based serial learning and proposed the working hypothesis that the processing mechanisms used for encoding serial order information that underlies the Hebb Effect are closely linked and associated with the same coding processes involved in ISR tasks such as digit span, non-word repetition, non-word paired associate learning as well as the phonological word-form learning component underlying lexical acquisition. All these processing tasks require the encoding and processing of item and serial order information and all of them rely on establishing links between the contents of short-term memory and representations of serial order information in long-term memory.

The recent literature on the Hebb Effect and its relationship to novel word form learning is extensive and growing rapidly and will not be reviewed here because of space limitations (see Mosse and Jarrold, 2008; Szmalec et al., 2009, 2012). However, there are a number of reasons why the HRL effect has become the focus of new research efforts on serial learning and memory, and it is worth mentioning them here. First, the topic of processing and encoding serial order information continues to be an important and central issue of research and theory in the field of cognition since the earliest days of experimental psychology and memory science going back to Ebbinghaus (1964) and the seminal observations of Lashley (1951) on the role of serial order coding in complex behavior. In addition, it has been widely assumed that the encoding and processing of serial order information is often a core foundational component of other more complex cognitive processes in other domains, especially language and motor behaviors. Second, the HRL effect is very robust and is relatively easy to obtain in different populations. Third, the effect represents the intersection of both short-term and long-term memory processes involving the transfer of information from short-term “activity-traces” in immediate memory to more permanent and stable “structural-traces” of item and order information in long-term memory which are thought to involve fundamentally different underlying neural systems. Fourth, the observed repetition effects and experimental methodology used in studying HRL encompasses both implicit and explicit memory and learning processes, two areas of memory research that are typically treated as separate processing domains. Finally, and perhaps most importantly, several researchers have argued that the HRL effect can serve as a laboratory-based analog for the real-world everyday language learning activities that are involved in phonological word-form learning and lexical acquisition of novel words by children learning language (Page and Norris, 2009a).

The sequence learning and serial order encoding results observed in the HRL paradigm have broader theoretical relevance and implications for language learning and development and individual differences in several clinical populations who may have delays and/or deficits in receptive and expressive language learning. For instance, several recent studies have reported long-term serial-order learning problems in children and adults with dyslexia suggesting that they may have a fundamental impairment in the encoding and processing of serial order information that results in weaker and more fragile lexical representations of words in long-term memory (see Szmalec et al., 2011; Bogaerts et al., 2015a,b). Other recent studies on adults with dyslexia have reported increases in proactive interference in an n-back task (Bogaerts et al., 2015b), suggesting that the deficit in serial order memory may also affect automatic inhibitory control processes used in verbal working memory tasks which commonly require the encoding and representation of both item and order information and the control of active attention.

The close parallels between these diverse sets of results obtained with several different populations and somewhat different experimental methodologies are probably not just a random coincidence. Instead, they very likely reflect a common domain-general core disturbance and/or impairment in the same basic underlying serial order coding processes that are involved in encoding, storing, and retrieving item and order information in verbal sequence memory and learning tasks. Our findings on Simon sequence memory and learning with deaf children who use CIs, along with a body of other results, reflect a deficit or disturbance/delay in the operation of a common serial order cognitive mechanism that is intimately involved in binding, chunking, and recoding repeated serial patterns reflecting the same processing operations used in sequence memory and novel word-form learning. We will return to these core issues again in the “Theoretical and Clinical Implications” Section of the paper.

## Implicit Learning of Sequential Patterns

Similar to the idea of the Hebb repetition effect, which demonstrates the learning of repeating patterns, is the notion of “statistical learning,” which reflects the acquisition of statistical-based regularities such as co-occurrence statistics or the probability of two stimuli occurring together in time or space. This type of statistical learning, a form of implicit learning, is currently thought to be one of the basic elementary learning mechanisms that is used in language acquisition (Cleeremans et al., 1998; Saffran et al., 2001; Altmann, 2002; Ullman, 2004). There are many studies on infants (Saffran et al., 1996), children (Meulemans et al., 1998), adults (Conway and Christiansen, 2005), and even non-humans (Petkov and Wilson, 2012) that have reported findings on implicit statistical pattern learning.

Several recent studies from our research group have explored the relations between individual differences in implicit statistical learning and spoken language processing abilities (Conway et al., 2007, 2010, 2011b; Conway and Pisoni, 2008; Shafto et al., 2012). In one of our initial studies, young NH adults carried out an implicit statistical learning task involving visual sequences and a sentence perception task that required listeners to recognize words in noise. The test sentences were taken from the Speech in Noise Test (SPIN) and varied on the predictability of the final word (Kalikow et al., 1977). We found that performance on the implicit learning task was correlated with performance on the speech perception task – specifically, for the high predictability SPIN sentences that had a highly predictable final word. This result was observed even after controlling for the variance associated with non-verbal intelligence, short-term memory, working memory, and attention and inhibition (see Conway et al., 2007, 2010).

The findings obtained with NH adults suggested that domain-general abilities related to implicit learning of sequential patterns are closely coupled with the ability to acquire and use information about the predictability of words occurring in degraded spoken sentences, knowledge that is critical for the successful acquisition of linguistic competence. The more experience that an individual has with the underlying sequential patterns of spoken language, the better one is able to use one’s long-term knowledge of those patterns to perceive and understand novel spoken utterances, and to reliably predict upcoming words in sentences, especially under degraded or challenging listening conditions. While our initial studies provided some preliminary evidence for an important empirical link between implicit learning and language processing in NH adults, in order to better understand the development of implicit learning, it is necessary to investigate implicit statistical learning processes in both typically developing and atypical clinical populations such as profoundly deaf children who have been deprived of sound and the typical environmental conditions of development that are appropriate for robust language learning.

Toward this end, we investigated implicit learning in a group of deaf children with CIs and a chronologically age-matched control group of NH typically developing children to assess the effects that a period of auditory deprivation and delay in language may have on learning of complex visual sequential patterns (Conway et al., 2011a). Some evidence already suggested that a period of auditory deprivation occurring early in development may have secondary cognitive and neural sequelae in addition to the obvious first-order hearing-related sensory effects (see Myklebust and Bruton, 1953; Luria, 1973; Conrad, 1979). Specifically, because sound is a physical signal distributed in time, lack of experience with sound patterns may affect how well a child is able to encode, process, and learn sequential patterns and encode and store temporal information in memory (Rileigh and Odom, 1972; Todman and Seedhouse, 1994; Fuster, 1995, 1997, 2001; Marschark, 2006). We have suggested that exposure to sound may also serve as a kind of “auditory scaffolding” in which a child gains specific experiences and practice with learning and manipulating sequential patterns in the environment (Conway et al., 2009, 2011b). Based on our earlier implicit visual sequence learning research with NH adults, we predicted that deaf children with CIs would show disturbances in visual implicit learning of sequential patterns because of their lack of experience with auditory temporal patterns early on in development. We also predicted that their implicit learning abilities would be associated with several measures of language development.

Two groups of 5–10 year-old-children participated in this study. One group consisted of 25 deaf children with CIs; the second group consisted of 27 age-matched typically developing, NH children. All children carried out an implicit visual sequence learning task. Several clinical measures of language outcome were also available for the CI children from our larger longitudinal study. Scores on these tests were also obtained for the NH children. Our specific hypothesis was that if some core foundational aspects of language development draw on domain-general learning abilities, then we should observe correlations between performance on the implicit visual sequence learning task and several different measures of spoken language processing. Measures of vocabulary knowledge and immediate memory span were also collected from all participants in this study in order to rule out obvious mediating variables that might be responsible for any observed correlations. The presence of correlations between implicit sequence learning and language processing even after partialing out the common sources of variance associated with these other measures would provide support for the hypothesis that implicit learning is directly associated with spoken language development, rather than being mediated by a third contributing factor.

Two artificial grammars (Grammars A and B) were used to generate the colored sequences used in the implicit learning task. These grammars specified the probability of a particular color occurring given the preceding color in sequence. Sequence presentation consisted of colored squares appearing one at a time, in one of four possible positions in a 2 × 2 matrix on a computer touchscreen in a manner that mimicked the basic design of the previous Simon memory game. The four states (1–4) of each grammar were randomly mapped onto each of the four screen locations as well as four possible colors (red, blue, yellow, green). The assignment of states in the grammar to position/color was randomly determined for each subject; however, for each subject, the mapping remained consistent across all trials. Grammar A was used to generate 16 unique sequences for the learning phase and 12 sequences for the test phase. Grammar B was used to generate 12 additional novel sequences for the test phase.

The children were told that they would see sequences of four colored squares displayed on the computer touch screen monitor. The squares would flash on the screen in a pattern and their job was to remember the pattern of colors on the screen and reproduce the sequence at the end of each trial by touching the square boxes on the computer monitor. The procedures for both the learning and test phases were identical and from the perspective of the subject, there was no indication of separate phases at all. The only difference between the two phases was which sequences were used. In the Learning Phase, the 16 learning sequences from Grammar A were presented first. After completing the reproduction task for all of the learning sequences, the experiment seamlessly transitioned to the Test Phase, which used the 12 novel sequences from Grammar A and the 12 novel Grammar B test sequences. The children were not told that there was an underlying grammar for any of the learning or test sequences or that there were two types of sequences in the Test Phase. The child just observed and then reproduced the visual sequences.

A sequence was scored correct if the child reproduced the entire test sequence correctly. Sequence span scores were then calculated using a weighted method in which the total number of correct test sequences at a given length was multiplied by the length and then scores for all lengths were added together (see Cleary et al., 2001). We calculated separate sequence span scores for Grammar A and Grammar B test sequences for each subject. We also calculated an implicit learning score for each subject, which was the difference in sequence span scores between the learned grammar (Grammar A) and the novel grammar (Grammar B). The implicit learning score measured generalization and reflected how well sequence memory spans improved for *novel* sequences that were constructed by the same grammar that subjects had previously experienced in the Learning Phase, relative to span scores for novel sequences created by Grammar B.

**FIGURE 4:**
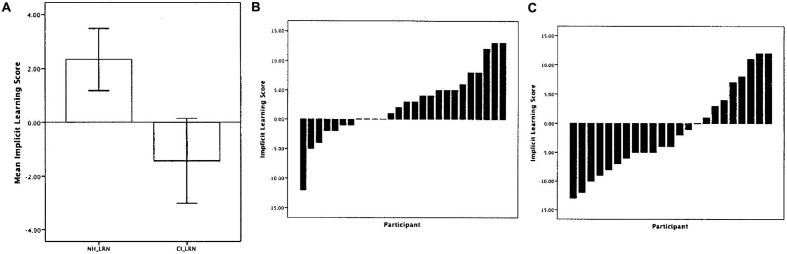
**Results from the visual implicit learning experiment for normal-hearing children and deaf children with CIs reported by Conway et al. (2010). (A)** The average visual implicit learning scores for the group of normal-hearing children (left) and the group of deaf children with CIs (right). **(B)** The implicit visual learning scores for each of the individual subjects in the normal-hearing group. **(C)** The implicit visual learning scores for each of the deaf children with CIs. [Adapted from results reported by Conway et al. (2010)].

**Figure 4** shows the average implicit learning scores for both groups of children (left). For the NH children, the average implicit learning score was 5.8% which was significantly greater than 0 demonstrating that as a group the NH children showed better learning of test sequences with the same statistical structure as the sequences from the initial Learning Phase. On the other hand, the average implicit learning score for the children with CIs was -2.5%, a value that was not statistically different from 0. In addition, the NH group’s implicit learning score was significantly greater than the CI group. **Figure 4** also shows the implicit learning scores for the individual children in the NH group (middle) and the CI group (right). Whereas 14 out of 26 (53.8%) of the NH children showed an implicit learning score of 0 or higher, only 8 out of 23 (34.7%) of the CI children showed a learning score above 0.

The present results demonstrate that deaf children with CIs show atypical implicit statistical learning of visual sequential patterns compared to age-matched NH children. This result is consistent with the hypothesis that a period of deafness and language delay may cause secondary disturbances in the development of sequencing skills. In addition, for the children with CIs, we computed a partial correlation between their implicit learning score and age at implantation, with chronological age partialed out. Implicit learning was negatively correlated with the age at which the child received their implant (*r* = -0.410, *p* = 0.058) and positively correlated with the duration of implant use (*r* = 0.410, *p* = 0.058). The longer the child was deprived of auditory stimulation, the lower the visual implicit learning scores; correspondingly, the longer the child had experience with sound via his/her implant, the higher the implicit learning scores. These correlations suggest that exposure to sound via a CI has secondary indirect effects on basic serial learning processes that are not directly associated with hearing, audibility, speech perception or language development; longer implant use appears to be associated with better ability to implicitly learn complex visual serial patterns and acquire knowledge about the underlying abstract grammar that generated the patterns.

Finally, in order to assess the association between implicit learning and language outcomes in the children with CIs, we computed bivariate correlations between the implicit learning score and three subtest scaled scores of the Clinical Evaluation of Language Fundamentals, fourth edition (CELF-4; Semel et al., 2003). These three subtests measure aspects of general language ability, including auditory comprehension, spoken sentence generation, and spoken sentence imitation. The implicit learning score was positively correlated with all three subtests, and for the most part this positive association remained significant even after controlling for the common variance associated with duration of implant use, forward digit spans, backward digit spans, and vocabulary scores.

In a related study, both groups of children also completed a sentence recognition task (Conway et al., 2014a,b), using the set of lexically controlled sentences developed by Eisenberg et al. (2002). The stimuli consisted of twenty lexically easy words (i.e., high word frequency, low neighborhood density) and twenty lexically hard words (i.e., low word frequency, high neighborhood density) embedded in short meaningful English sentences. The sentences were presented over a loudspeaker at 65 dB SPL. The children were instructed to listen closely to each sentence and then repeat back what they heard to the examiner even if they were only able to perceive one word of the sentence. All of the test sentences were presented in random order to each child. Responses were recorded onto digital audiotape and were later scored off-line based on number of keywords correctly repeated for each sentence. The sentences were played in the quiet without any degradation to the deaf children with CIs. For the NH children, the original sentences were spectrally degraded to simulate a CI using a four-channel sinewave vocoder to reduce performance from ceiling levels (Shannon et al., 1995).

For both groups, performance was analyzed for recognition accuracy of each of the three key words in each sentence. This allowed us to examine the extent that the children were using sentence context to improve their perception and reproduction of the spoken words. Whereas the NH children showed robust effects of contextual facilitation as measured by improved performance for the third word in each sentence compared to the first word, the deaf children with CIs on average showed no such contextual facilitation. When taken together with our previous findings with NH adults showing that better implicit serial learning abilities result in more robust knowledge of the sequential predictability of words in sentences which leads in turn to more efficient use of sentence context to aid spoken word recognition processes (Conway et al., 2010), it is possible that the deaf children’s inability to make use of sentence context is due to their observed disturbances to implicit learning.

In sum, these recent studies showed that the deaf children with CIs display atypical implicit learning abilities, possibly due to a lack of early experience with auditory patterns and/or exposure to spoken language. Implicit sequence learning abilities in turn were positively correlated with better language scores even after controlling for other general cognitive scores. Finally, we found that these children displayed an inability to use sentence context to facilitate the perception of spoken words, possibly as a consequence of their disturbances in implicit sequence learning. It appears that these children were treating sentences as “strings of unrelated words” (Eisenberg et al., 2002; Conway et al., 2014a), not having a good sense of how various words co-occur with each other in a given sentence context and being unable to use previous words and prior supporting context to help them perceive and recognize subsequent words.

## Verbal Learning and Memory Processes

Although we are now beginning to make some significant progress in understanding how normal-hearing listeners manage to recognize and understand speech under many adverse and challenging conditions and how they carry this process out so quickly and efficiently, very little basic or clinical research has focused on investigations of the underlying processes responsible for rapid adaptation, adjustment and perceptual learning in hearing impaired listeners who use CIs. Most of the outcomes research on speech perception and sentence recognition skills in CI-users has been carried under benign testing conditions in quiet in the audiology clinic or laboratory using conventional low-variability test materials (words and sentences) that place very few, if any, processing demands on rapid automatized processes such as perceptual adaptation, adjustment or normalization. To the best of our knowledge, no studies have investigated the elementary foundational processes related to verbal learning and memory processes in this clinical population.

Fundamental questions about the nature of rapid perceptual adaptation and perceptual normalization and issues dealing with how CI users process compromised underspecified acoustic signals have not been fully investigated in this clinical population despite many years of research on outcomes. This is not surprising because only a small handful of studies have been carried out on working memory dynamics (capacity, speed, updating, inhibition, shifting, switching, etc.), and long-term episodic, procedural and semantic memory processes that underlie robust speech recognition and spoken language processing in normal hearing listeners. The available evidence from several recent studies strongly suggests that rapid adaptation, robust perceptual adjustment and normalization to multiple sources of variability in the speech signal is critically dependent on a small set of neurocognitive factors– elementary processes related to learning and memory, attention, inhibition, executive functioning, and cognitive control processes.

Learning is fundamental to all adaptive behaviors in living organisms and is inseparable from the sensory, perceptual, and cognitive processes involved in the acquisition, storage, and retrieval of information in long-term memory. The fluency and perceptual robustness routinely observed in processing speech signals under challenging conditions by normal hearing listeners reflects the operation of the entire information processing system working together in an integrated fashion (Oblesser et al., 2007). No single component taken alone in isolation from the rest of the processing system is entirely responsible for the observed robustness and perceptual integrity of the final product of the comprehension process– successfully recovering the talker’s intended linguistic message. While there can be little doubt that basic elementary learning and memory processes play a fundamental role in the development of speech and language and perceptual adaptation and normalization skills in challenging listening conditions, this foundational topic in cognition has been seriously neglected in the field of hearing loss and, specifically, in the field of CIs.

To begin studying the elementary cognitive factors and information processing operations that underlie robust speech perception and spoken word recognition skills, we have significantly broadened the conventional end-point product-based approach typically used in assessing outcomes and individual differences in CI-users by directly investigating basic fundamental verbal learning and memory processes in pre-lingually deaf CI users. In a recent study, we obtained some preliminary results using a well-known norm-referenced neuropsychological test of verbal learning and memory, the CVLT-II, which has been used extensively with several different clinical populations although it has not been used with prelingually deaf long-term CI-users. Only one other study has used the CVLT with hearing-impaired listeners. Heydebrand et al. (2007) administered the CVLT-II before implantation to a group of 44 post-lingually deaf adults who were candidates for cochlear implantation in order to predict their audiological and speech recognition outcomes 6 months after surgery. They found that a composite verbal learning score based on four CVLT sub-scores was a strong predictor (*r* = 0.82) of CNC speech recognition scores post-CI after controlling for CNC speech recognition at baseline before implantation. Their results suggest that verbal learning may play a central foundational role in speech and language outcomes following implantation because basic learning processes share common variance with the information processing tasks routinely used to measure speech perception and spoken language understanding.

The CVLT makes use of a multi-trial free recall (MTFR) methodology to obtain measures of several foundational cognitive processes used in verbal learning and memory such as repetition-based multi-trial free recall, primacy and recency, proactive and retroactive interference, memory decay in free- and cued-recall and organizational strategies in memory retrieval such as serial, semantic, and subjective clustering that are often routinely used by subjects to make items more accessible for retrieval in free recall tasks (Delis et al., 2000). In the MTFR procedure used in the CVLT-II, subjects are read a list of 16 familiar words (List A) five times to measure repetition learning processes and free recall. The 16 words on List A were selected from four semantic categories. After each list is presented, the subject is asked to recall as many of the study items from List A as possible in any order. This free recall procedure is followed for five learning trials with List A. Each presentation involves one repetition of List A followed by free recall of the List A items. After the fifth presentation and recall of List A items, subjects are presented with a new list of 16 words, List B, to measure proactive interference. List B also contains words from four semantic categories. After recall of List B, subjects are then asked to recall List A again (short-delay free recall) to measure retroactive interference produced by List B. Following a 20 min delay period during which the subject is engaged in a distractor task, the subject is asked to recall the words from List A again (long-delay free recall) to measure memory decay after a long delay interval.

The CVLT is a “high-yield” clinical test of verbal learning and memory processes that was designed to study repetition and organizational strategies used in free recall tasks. It produces a large amount of clinically relevant data in a short assessment time. The scores obtained from the CVLT provide important diagnostic information about basic core verbal learning and memory processing skills that are related to domains of executive functioning and cognitive control such as controlled attention, fluency-speed, abstraction, self-generated retrieval organization strategies and mental control processes as well as spoken word recognition, encoding, storage and retrieval strategies.

**FIGURE 5:**
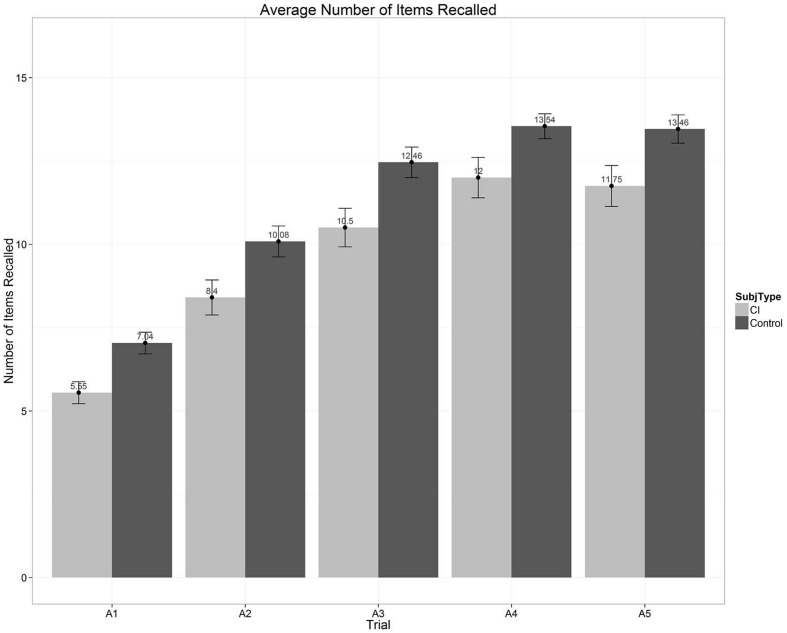
**Average number of words recalled as a function of the five repetition learning trials for List A on the CVLT-II.** Scores for the prelingually deaf CI users are shown by the light gray bars on the left; scores for the normal-hearing age- and IQ-matched normal-hearing controls are shown by the dark gray bars on the right.

**Figure 5** shows a global overall summary of the multi-trial free recall scores for the five repetitions of List A obtained from two groups of subjects that we tested recently (see Chandramouli et al., Manuscript in preparation for further details). The left set of bars in **Figure 5** show average free recall scores from a group of 20 prelingually deaf long-term CI users; the right set of bars shows the scores from a group of 24 normal-hearing controls who were matched in age and non-verbal IQ to the CI users. Both groups of subjects were part of a large ongoing research project dealing with executive function and cognitive control processes in long-term prelingual CI-users (Kronenberger et al., 2013, 2014). Each bar in **Figure 5** represents the average correct recall scores over the 16 items in each presentation of List A.

Inspection of **Figure 5** shows two main findings. First, both groups of subjects display robust repetition learning effects over the five presentations of List A. Second, the group of CI users shows consistently poorer total free recall scores after each repetition of List A compared to the NH controls. Looking only at the overall average measures of free recall performance shown in **Figure 5**, however, provides an incomplete picture of the underlying organizational and processing strategies that subjects use in carrying out this MTFR task with categorized word lists. In addition to providing total recall scores summed across all serial positions following the five repetitions of List A, the CVLT-II provides several other more detailed measures of verbal learning and memory processes obtained from separate analyses of the subcomponents of the serial position curve. Below we provide a brief summary of these findings, including (1) primacy and recency effects; (2) recall patterns and retrieval processes; (3) organizational strategies and semantic clustering; and (4) correlations with speech and language outcomes.

**FIGURE 6:**
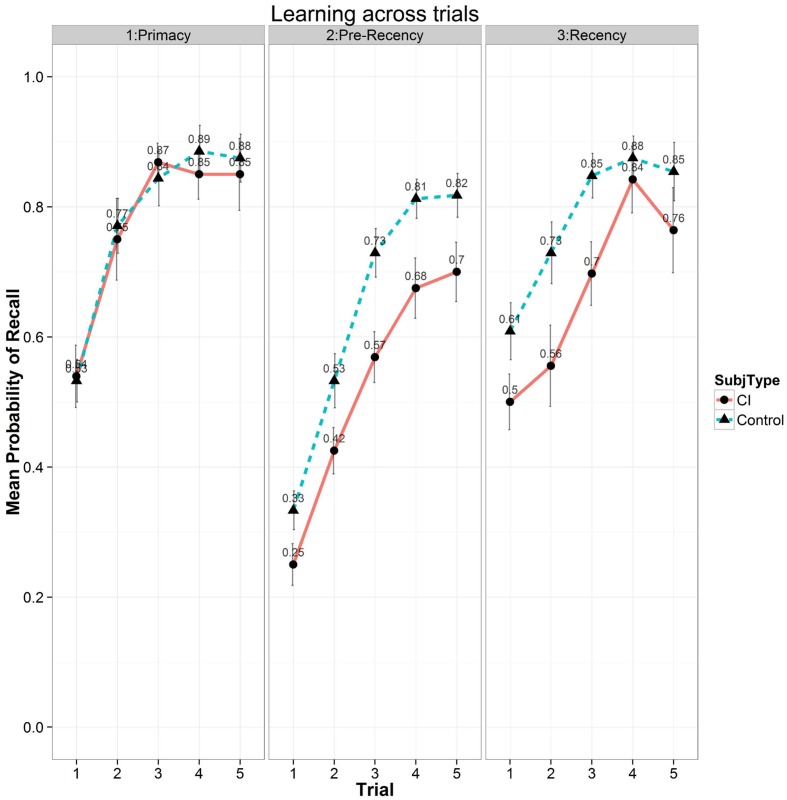
**Mean probability of correct recall as a function of the five repetition learning trials for List A on the CVLT-II for the three subcomponents of the conventional serial position curve.** Scores for the primacy portion of the serial position curve are shown on the **(Left)**, scores for the pre-recency (middle) position of the serial position curve are shown in the **(Center)**, and scores for the recency portion are shown on the **(Right)**. Results for the CI users are shown by solid bars and circles; results for the normal-hearing controls are shown by dashed bars and triangles.

In terms of primacy and recency effects, **Figure 6** shows a summary of the free recall scores as a function of the five List A repetitions for the three subcomponents, primacy (first four items), pre-recency (middle eight items) and recency (last four items) portions of the conventional serial position curve. These subcomponents are thought to reflect fundamentally different storage and retrieval processes in carrying out free recall tasks (Atkinson and Shiffrin, 1971). Recall scores from the primacy portion of the serial position curve are shown in the left-hand panel, scores from the pre-recency (middle) portion are shown in the center panel, and scores from the recency portion are shown in the right-hand panel of **Figure 6**. Examination of **Figure 6** shows two patterns in free recall. First, free recall consistently improves for both groups of subjects in all three subcomponents of the serial position curve following each of the five repetitions of List A. Second, the differences observed in free recall between the two groups are not comparable across all three subcomponents of the serial position curve but are confined selectively to only the pre-recency and recency portions of the serial position curve as shown in the center and right-hand panels. It should be noted that study items on the CVLT-II, List A are always presented in the same order during the MTFR phase. The absence of any differences between the two groups in the primacy portion of the serial position curve suggests that early list items were successfully encoded and retrieved equivalently by both groups of subjects. In contrast, the differences observed between the two groups in the pre-recency and recency portions suggest disturbances in the component processing operations used in verbal rehearsal and retrieval strategies possibly reflecting weaknesses in active rehearsal and transfer of incomplete or underspecified phonological and lexical representations of the list items. These differences may also reflect the use of different retrieval strategies as well.

**FIGURE 7:**
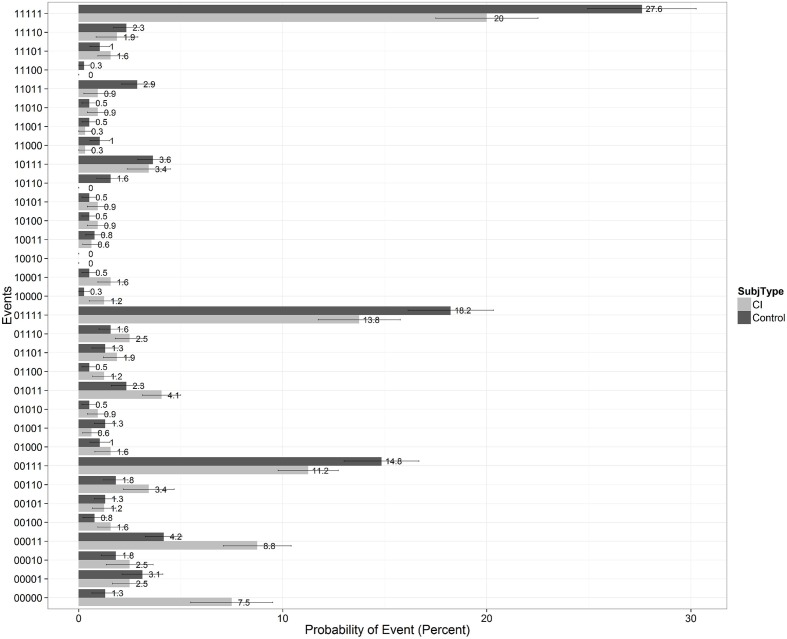
**Recall patterns showing the probability of the 32 possible recall-events collapsing across all subjects and items for the five learning trials of List A on the CVLT-II.** Scores for the CI users are shown by the light gray bars on the right of each column; scores for the normal-hearing controls are shown by the black bars on the left of each column. A “00000” denotes that a specific test item was never recalled on any of the five learning trials of List A; a “11111” denotes that a specific test item was correctly recalled on every trial, and so on.

To gain further insights into the recall patterns and retrieval processes, we visualized the data as shown in **Figure 7**. On any given learning trial, there are two possible states of recall for a specific test item: the item is either recalled or not recalled by the participant. Over the five repetitions of List A, there are 32 possible ways an item can be recalled. Following Batchelder et al. (1997), we called each of these possibilities a “recall-event” and computed the frequency distribution of recall-event occurrences for the two groups after collapsing over subjects and items on List A. The results of this analysis are shown in **Figure 7** where each of the 32 possible recall-events is listed on the ordinate and denoted by a 5-bit binary string in which each bit represents a correct recall or failure to recall an item on any given learning trial. Thus, a “00000” denotes that the item was never recalled on any of the five learning trials, a “00111” denotes that an item was not recalled on Trials 1 and 2 but was recalled on Trials 3, 4, and 5, and a “11111” denotes that an item was recalled on every trial, and so on for the remaining recall-events. The probability of each of the 32 recall-events is displayed on the abscissa separately for each of the two groups of subjects.

This recall-event analysis provides useful information about the temporal processing dynamics of item recall during repetition learning of items on List A. In **Figure 7**, we observed peaks at recall events such as “11111,” “01111,” “00111,” and “00011” which suggests that, on the whole, once encoded and learned, items tend to be recalled in each trial. We also observe in **Figure 7** a peak at “00000” for CI subjects. This means that the CI users were much more likely than controls to have never recalled an item over the five repetition learning trials (event ‘00000’). CI users had a 7.5% probability of never recalling an item (a small portion of which is caused due to misheard intrusions), and controls displayed only a 1.3% chance of never recalling an item. In addition, the CI users also required more trials before successfully recalling an item for the first time (i.e., more events where there are zero bits preceding the first occurrence of ‘1’ bit). Given that we are dealing with a group of subjects who may have inherent differences in audibility and resolution of the fine acoustic-phonetic details of speech inputs, it would be safe to say that many of the observed differences may come about due to differing ease of early sensory encoding or item registration between the two groups. However, the CI users were at ceiling on a separate auditory identification task using all of the test items in the two lists at the end of the CVLT test protocol. Moreover, all of the test items on both List A and List B were administered in the quiet. When we did an item analysis of the error responses, we did not observe any particular word or words that accounted for this trend.

While the previous finding about differences in encoding might not be surprising to many, what was interesting to us was the observation that given an item was recalled once, the CI users were more likely (25.85%) than controls (20.39%) to miss an item on the next trial. In carrying out this analysis, we considered only recall-events where an item was recalled at least once within the first four trials before calculating this percentage, and then we analyzed how likely they were to fail on future trials. Finding differences in these recall patterns in CI users compared to the controls suggests retrieval differences, especially if all-or-none models of encoding and retrieval are assumed. This analysis followed earlier efforts by Batchelder and Riefer (1986), Batchelder et al. (1997) and Riefer et al. (2002) who have used Multinomial Processing Tree (MPT) models to quantitatively estimate underlying process-measures using such recall-event patterns. We report just the qualitative results here for now using percentages and leave more accurate and quantitative estimates of encoding and retrieval probabilities by generating MPT models to future work. Chandramouli et al. (Manuscript in preparation) provide additional converging evidence that it is indeed the case that retrieval differences also exist between the groups. We suggest that these differences can be traced back to differences in the long-term developmental histories and early experiences and language processing activities of the two groups.

**FIGURE 8:**
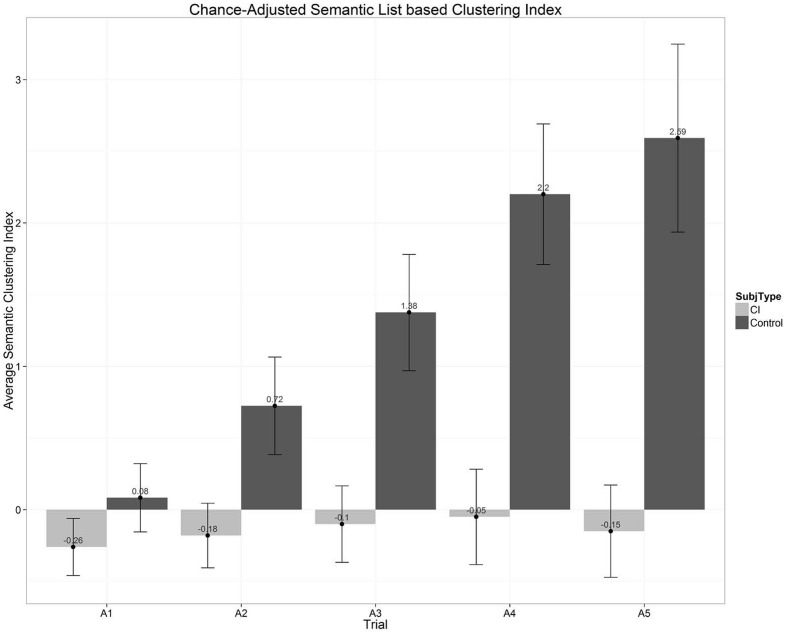
**Chance-adjusted semantic clustering scores as a function of the five repetition learning trials on List A of the CVLT-II.** The scores displayed in this figure show the difference between the number of observed clusters in the response protocol and the number of response clusters expected by chance for the observed total recall length. A positive difference indicates that the observed semantic clustering is greater than would be expected by chance. Scores for the CI users are shown in the gray bars; scores for the normal-hearing controls are shown by the black bars.

Next, we explored participants’ organizational strategies and semantic clustering. In free-recall or list-learning experiments using categorized word lists, the order in which participants recall items can be used to infer the type of organizational strategies that are used during encoding and retrieval. A semantic organizational strategy is observed when there is a higher probability of recalling a sequence of items in succession that are from the same semantic category. The CVLT-II quantifies this value by using a list-based semantic clustering index (Delis et al., 2000; Stricker et al., 2002). To obtain this measure, first the number of response clusters observed in each trial is tallied: whenever there is a correct recall followed immediately by another correct recall, and both are from the same semantic category, the tally increases by one. The chance-adjusted semantic clustering indices for the two groups of subjects are plotted in **Figure 8**. The semantic clustering index displayed here is a simple difference between the number of observed clusters and the number of clusters expected by chance for the observed total recall length. A positive difference indicates that the observed semantic clustering is higher than that expected by chance. In **Figure 8**, we can clearly see that the NH controls are using semantic strategies for organizing items in their memory. Moreover, their use of semantic clustering increases every successive trial even after the number of items recalled by the group stops increasing as they approach ceiling. In contrast, however, the use of semantic clustering strategies by the CI users remains at chance across the five learning trials. While a part of this result has to do with the higher incidence of intrusion errors that reduce the number of clusters observed for the CI-group, the results clearly show that the CI users as a group use semantic clustering much less often than the NH controls.

Finally, we investigated correlations with speech and language outcomes. In addition to the MTFR measures of verbal learning and memory using the CVLT-II, as part of a large project on individual differences in outcomes following long-term CI use, we also administered a battery of speech and language and executive function measures to assess the strengths, weaknesses, and milestones in these two groups of subjects (see Kronenberger et al., 2014). Measures of speech and language included conventional tests of receptive vocabulary, open-set spoken word recognition, sentence perception, non-word repetition as well as several indexical processing tasks such as regional dialect categorization and non-native speaker ratings. Measures of executive functioning included neuropsychological tests such as digit span, Stroop color-word naming, number–letter switching, retrieval fluency, coding copy, visual matching, and concept formation. To investigate the relations between a subset of measures obtained from the CVLT-II (total words correctly recalled, learning slope over the five List A repetition trials and the average semantic clustering index) and the speech and language and executive function scores, we carried out a series of simple bivariate correlations. CVLT total words recalled correlated significantly (*p* < 0.05) with DKEFS number–letter switching (*r* = –0.50), Stroop color-word naming (*r* = 0.46) and WISC coding (*r* = 0.50). CVLT learning slope was correlated with fragmented visual sentence recognition (*r* = 0.61) and non-word repetition of syllables (*r* = 0.45). CVLT average semantic clustering was correlated with Stroop color word naming (*r* = 0.38), non-word repetition (*r* = 0.42), and recognition of keywords in foreign-accented PRESTO sentences (*r* = 0.37). These initial findings provide converging evidence of associations between measures of verbal learning and memory obtained from the CVLT-II and measures of executive functioning and speech perception in long-term CI users suggesting that the same elementary information processing operations are shared by all these sets of measures.

## Theoretical and Clinical Implications

Some profoundly deaf children with CIs do extremely well on traditional clinically based speech and language outcome measures while other children have much more difficulty after they receive their CIs. The enormous variability in outcome and benefit following cochlear implantation is recognized as a significant clinical problem in the fields of pediatric hearing loss, otology, and clinical audiology, although it has not received adequate attention by clinicians or research scientists working on CI outcomes in the past. Until we are able to obtain a much better understanding of the underlying early sensory and cognitive basis of individual differences in outcomes, we will continue to face significant challenges in developing new approaches for diagnosis, treatment, and especially the early identification of deaf children who may be at high risk for poor speech and language outcomes after implantation. New fundamental knowledge about the underlying elementary sensory and cognitive processes that contribute to the observed variability in speech and language outcomes will also play an important role in developing novel robust interventions following implantation in terms of selecting specific methods for habilitation and treatment that are specifically targeted for an individual child based on his or her strengths, weaknesses and milestones. We have now identified the locus of two areas of weakness in the neurocognitive functioning that may underlie variability in speech and language outcomes: (1) basic domain-general learning abilities, specifically, explicit and implicit serial learning; and (2) the organizational processes and retrieval strategies used in verbal learning and memory in free recall of categorized lists of spoken words. The new findings on organizational processes in free recall of categorized word lists obtained from the CVLT-II suggest that semantic clustering strategies are significantly compromised in long-term CI users who show little evidence of making efficient use of semantic similarity relations among words to facilitate retrieval of items from long-term memory.

Many deaf children with CIs may have other comorbidities and/or disturbances in basic neurocognitive processes that serve as the foundation for the information processing systems used in spoken language processing. These comorbidities and disturbances appear to be, at least in part, secondary to their profound hearing loss and delay in language development (Conrad, 1979). A period of auditory deprivation during critical developmental periods before implantation affects sensory and cognitive development in a variety of ways (Luria, 1973). Differences resulting from both deafness and subsequent neural reorganization and plasticity of multiple brain systems may therefore be responsible for the enormous variability observed in speech and language outcome measures following implantation. Without knowing what specific underlying neurobiological and neurocognitive factors are responsible for the individual differences in speech and language outcomes, it is difficult to recommend an appropriate and efficacious approach to habilitation and speech-language therapy after a child receives a CI. More importantly, the deaf children who are performing poorly with their CIs are not a homogeneous group and may differ in numerous ways from one another, reflecting dysfunction of multiple brain systems associated with congenital deafness and profound hearing loss. From a clinical perspective, it seems very unlikely that an individual child will be able to achieve optimal speech and language benefits from his/her CI without knowing why the child is having speech and language problems and which particular neurocognitive domains underlie these problems.

In addition to the earlier findings reported in this paper on explicit and implicit sequence learning and memory processes using the Simon memory game and the new more recent results obtained on verbal learning and memory using the CVLT-II to study multi-trial free recall strategies, we have also carried out a number of other studies over the past 15 years on the ISR skills of deaf children with CIs using traditional measures of digit span as well as novel measures of non-word repetition, talker discrimination, and regional dialect categorization (Tamati and Pisoni, 2015). Although all of these behavioral tasks use quite different experimental procedures and methodologies and measure somewhat different information processing skills when looked at superficially, there are several elementary components in common across these tasks that provide some important new insights into the underlying processing architecture and mechanisms that appear to be responsible for the delays and deficits observed in speech and language and executive functioning in this clinical population. When all of our findings are considered together, a consistent pattern begins to emerge suggesting a process-based explanation for the differences observed between deaf children with CIs and age-matched NH children and for the individual differences and variability observed in outcomes. This processing-based account is mechanistic in nature involving the rapid encoding of item and order information in speech and episodic context of the encoding conditions.

One of the most important and critical components underlying speech and language processing is the early encoding, storage, and use of item and order information and episodic contexts in representations and processing of spoken language (Page and Norris, 2009a). Regardless of whether we are considering word recognition, sentence perception or comprehension of sequences of meaningful sentences, sequencing and the episodic encoding of item and order information is central to all aspects of spoken language processing. We propose that the initial registration and processing of item and order information and encoding of episodic context is significantly compromised in this clinical population (Conway et al., 2009) and that this domain-general impairment in basic sequential processing skills creates cascading effects on later higher-order speech and language processing operations used in rapid phonological coding, word recognition, lexical access, verbal retrieval, syntactic parsing, and comprehension. Deficits in registration and early encoding of the episodic context and fine acoustic-phonetic details of speech are observed across the board in a wide range of different language processing tasks including open-set word recognition, sentence recognition in quiet and noise and non-word repetition as well as indexical processing tasks such as talker discrimination and recognition, regional dialect classification and judgments of speech quality and speech intelligibility. All of these processes rely on the registration, early encoding, storage, retrieval and processing of highly detailed memory representations that preserve item and order information in sequential patterns. Although we only have some intuitions and tentative hypotheses at this time, we believe it is very likely that the core deficits in all of these ISR tasks may reflect more basic elementary impairments and deficits in the fine episodic encoding of context and environmental conditions at the time of acquisition which attenuate and often prevent the efficient registration of highly detailed phonetic and sublexical representations of spoken words in isolation and in sentences.

Much of the clinical research carried out on CIs since they became widely adopted as the standard of care for profoundly deaf children has been intellectually isolated from the mainstream of current research and theory in the fields of neuroscience, cognitive psychology and developmental neuropsychology. As a consequence, the major clinical research issues have been very narrowly focused on speech and language outcomes and efficacy of cochlear implantation as a medical treatment for profound hearing loss. Relatively little basic or clinical research has investigated the elementary information processing operations and components—the building blocks of cognition that underlie the enormous individual differences and variability routinely observed in measures of the effectiveness of CIs. Moreover, very few studies have attempted to identify early neurocognitive predictors of outcome and benefit or to systematically assess the effectiveness of specific neurocognitive interventions or habilitation strategies after implantation. As discussed earlier, although variables like age of implantation, communication mode, family and device factors, and various audiological and hearing-related variables clearly play an important role in understanding the nature of variation in speech and language outcomes, we believe that these factors alone are only part of the story. Additional sources of variance, such as those arising from basic processes of learning, memory, and cognition, are needed to fully understand the underlying mechanisms that contribute to successful speech and language outcomes following cochlear implantation.

We believe these are important new directions for clinical research on CIs in the future, directions that draw heavily on basic research, theory, and methodology in the fields of cognition and cognitive science that represent the intersection of several closely related scientific disciplines that are all concerned with brain plasticity, neural development, learning and memory, attention, executive function and cognitive control. As Carol Flexer observed a few years ago, “Hearing loss is primarily a brain issue, not an ear issue,” (Flexer, 2011). Until we begin to recognize the important down-stream contributions of central auditory and cognitive factors and the role of the entire information processing system working together, researchers working on CIs will continue to carry out the same kind of conventional outcome studies expecting different results that will never lead to new advances in evidence-based interventions for deaf children who are doing poorly with their CIs.

## Author Contributions

DP wrote the first draft; WK edited and rewrote several sections; SC carried out the research reported in the “Explicit Sequence Memory Spans” Section; CC edited and rewrote several sections and was the lead researcher for the studies reported in the “The Puzzle about Outcomes following Cochlear Implantation” Section.

## Conflict of Interest Statement

The authors declare that the research was conducted in the absence of any commercial or financial relationships that could be construed as a potential conflict of interest.
